# A framework to evaluate research capacity building in health care

**DOI:** 10.1186/1471-2296-6-44

**Published:** 2005-10-27

**Authors:** Jo Cooke

**Affiliations:** 1 Primary Care and Social Care Lead, Trent Research and Development Unit, formerly, Trent Focus Group, ICOSS Building, The University of Sheffield, 219 Portobello, Sheffield S1 4DP, UK

## Abstract

**Background:**

Building research capacity in health services has been recognised internationally as important in order to produce a sound evidence base for decision-making in policy and practice. Activities to increase research capacity for, within, and by practice include initiatives to support individuals and teams, organisations and networks. Little has been discussed or concluded about how to measure the effectiveness of research capacity building (RCB)

**Discussion:**

This article attempts to develop the debate on measuring RCB. It highlights that traditional outcomes of publications in peer reviewed journals and successful grant applications may be important outcomes to measure, but they may not address all the relevant issues to highlight progress, especially amongst novice researchers. They do not capture factors that contribute to developing an environment to support capacity development, or on measuring the usefulness or the 'social impact' of research, or on professional outcomes.

The paper suggests a framework for planning change and measuring progress, based on six principles of RCB, which have been generated through the analysis of the literature, policy documents, empirical studies, and the experience of one Research and Development Support Unit in the UK. These principles are that RCB should: develop skills and confidence, support linkages and partnerships, ensure the research is 'close to practice', develop appropriate dissemination, invest in infrastructure, and build elements of sustainability and continuity. It is suggested that each principle operates at individual, team, organisation and supra-organisational levels. Some criteria for measuring progress are also given.

**Summary:**

This paper highlights the need to identify ways of measuring RCB. It points out the limitations of current measurements that exist in the literature, and proposes a framework for measuring progress, which may form the basis of comparison of RCB activities. In this way it could contribute to establishing the effectiveness of these interventions, and establishing a knowledge base to inform the science of RCB.

## Background

The need to develop a sound scientific research base to inform service planning and decision-making in health services is strongly supported in the literature [1], and policy [2]. However, the level of research activity and the ability to carry out research is limited in some areas of practice, resulting in a low evidence base in these areas. Primary Care, for example, has been identified as having a poor capacity for undertaking research [3-5], and certain professional groups, for example nursing and allied health professionals, lack research experience and skills [5-7]. Much of the literature and the limited research on research capacity building (RCB) has therefore focused on this area of practice, and these professional groups. Policy initiatives to build research capacity include support in developing research *for *practice, where research is conducted by academics to inform practice decision making, research *within or through *practice, which encompasses research being conducted in collaboration with academics and practice, and research *by *practice, where ideas are initiated and research is conducted by practitioners [3,8].

The interventions to increase research capacity for, within, and by practice incorporates initiatives to support individuals and teams, organisations and networks. Examples include fellowships, training schemes and bursaries, and the development of support infrastructures, for example, research practice networks [9-13]. In the UK, the National Coordinating Centre for Research Capacity Development has supported links with universities and practice through funding a number of Research and Development Support Units (RDSU) [14]which are based within universities, but whose purpose is to support new and established researchers who are based in the National Health Service (NHS). However, both policy advisers and researchers have highlighted a lack of evaluative frameworks to measure progress and build an understanding of what works[15,16].

This paper argues for a need to establish a framework for planning and measuring progress, and to initiate a debate about identifying what are appropriate outcomes for RCB, not simply to rely on things that are easy to measure. The suggested framework has been generated through analysis of the literature, using policy documents, position statements, a limited amount of empirical studies on evaluating research RCB, and the experience of one large RSDU based in the UK.

## Discussion

The Department of Health within the UK has adopted the definition of RCB as *'a process of individual and institutional development which leads to higher levels of skills and greater ability to perform useful research". (pp1321) *[17]

Albert & Mickan cited the National Information Services in Australia [18] who define it as

" an approach to the development of sustainable skills, organizational structures, resources and commitment to health improvement in health and other sectors to multiply health gains many times over.'

RCB can therefore be seen as a means to an end, the end being 'useful' research that informs practice and leads to health gain, or an end in itself, emphasising developments in skills and structures enabling research to take place.

A framework for measuring capacity building should therefore be inclusive of both process and outcome measures [19], to capture changes in both the 'ends' and 'means'; it should measure the ultimate goals, but also measure the steps and mechanisms to achieve them. The notion of measuring RCB by both process and outcome measures is supported within the research networks literature [12,20], and capacity building in health more generally [19,21]. Some argue we should acknowledge 'process as outcome', particularly if capacity building is seen as an end in itself [21]. In this context process measures are 'surrogate' [12], or 'proxy' outcome measures[16]. Carter et al [16]stress caution in terms of using 'proxy' measures in the context of RCB, as there is currently little evidence to link process with outcome. They do not argue against the notion of collecting process data, but stress that evaluation work should examine the relationship of process to outcome. The proposed framework discussed in this paper suggests areas to consider for both process and outcome measurement.

The most commonly accepted outcomes for RCB cited in the literature includes traditional measures of high quality research including publications, conference presentations, successful grant applications, and qualifications obtained. Many evaluations of RCB have used these as outcomes [9,10,22,23]. Some argue that publications in peer reviewed journals are a tall order for the low research skills base in some areas of health care practice [5], and argue for an appropriate time frame to evaluate progress. Process measures in this context could measure progress more sensitively and quickly.

However, using traditional outcomes may not be the whole story in terms of measuring impact. Position statements suggest that the ultimate goal of research capacity building is one of health improvement [17,18,24]. In order for capacity building initiatives to address these issues, outcomes should also explore the direct impact on services and clients: what Smith [25]defines as the social impact of research.

There is a strong emphasis within the primary care literature that capacity building should enhance the ability of practitioners to build their research skills: to support the development of research 'by' and 'with' practice [3,26], and suggests 'added value' to develop such close links to practice. A framework to measure RCB should explore and try to unpack this 'added value', both in terms of professional outcomes,[10] which include increasing professional enthusiasm, and supporting the application of critical thinking, and the use of evidence in practice. Whilst doing research alongside practice is not the only way these skills and attitudes can be developed, it does seem to be an important impact of RCB that should be examined.

The notion of developing RCB close to practice does not necessarily mean that it is small scale just because it is close to the coal face. Obviously, in order for individuals and teams to build up a track record of experience their initial projects may justifiably be small scale, but as individual's progress, they may gain experience to be able to conduct large scale studies, still based on practice problems, working in partnership with others. Similarly networks can support large scale studies as their capacity and infrastructure is developed to accommodate them.

### The framework

**Figure 1:**
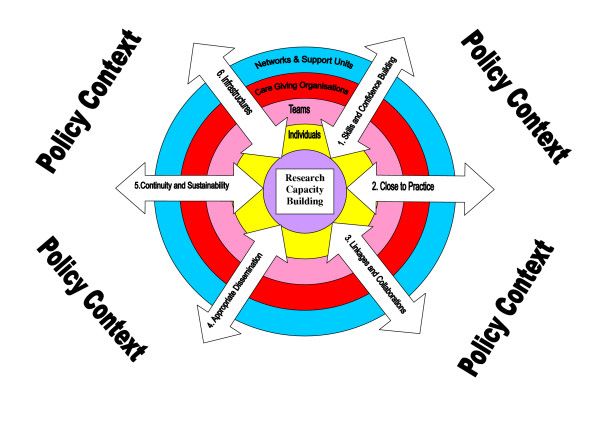
Research Capacity Building: A Framework for Evaluation.

The framework is represented by Figure 1. It has two dimensions

• **Four structural levels of development activity**. These include individual, team, organisational, and the network or supra- organisational support level (networks and support units). These are represented by the concentric circles within the diagram.

• **Six principles of capacity building**. This are discussed in more detail below but include: building skills and confidence, developing linkages and partnerships, ensuring the research is 'close to practice', developing appropriate dissemination, investments in infrastructure, and building elements of sustainability and continuity. Each principle is represented by an arrow within the diagram, which indicates activities and processes that contribute towards capacity building. The arrows cut across the structural levels suggesting that activities and interventions may occur within, and across, structural levels. The arrow heads point in both directions suggesting that principles applied to each structural level could have an impact on other levels.

The framework acknowledges that capacity building is conducted within a policy context. Whilst this paper focuses on measurement at different structural levels, it should be acknowledged that progress and impact on RCB can be greatly nurtured or restricted by the prevailing policy. Policy decisions will influence opportunities for developing researchers, can facilitate collaborations in research, support research careers, fund research directed by practice priorities, and can influence the sustainability and the very existence of supportive infrastructures such as research networks.

The paper will explain the rationale for the dimensions of the framework, and then will suggest some examples of measurement criteria for each principle at different structural levels to evaluate RCB. It is hope that as the framework is applied, further criteria will be developed, and then used taking into account time constraints, resources, and the purpose of such evaluations.

### Structural levels at which capacity building takes place

The literature strongly supports that RCB should take place at an individual and organisational level [8,15,27,28]. For example, the conceptual model for RCB in primary care put forward by Farmer & Weston [15] focuses particularly on individual General Practitioners (GPs) and primary care practitioners who may progress from non participation through participation, to become academic leaders in research. Their model also acknowledges the context and organisational infrastructure to support RCB by reducing barriers and accommodating diversity through providing mentorship, collaborations and networking, and by adopting a whole systems approach based on local need and existing levels of capacity. Others have acknowledged that capacity development can be focussed at a team level [11,29]. Jowett et al [30] found that GPs were more likely to be research active if they were part of a practice where others were involved with research. Guidance from a number of national bodies highlights the need for multiprofessional and inter-professional involvement in conducting useful research for practice [3,4,6,31] which implies an appropriate mix of skills and practice experience within research teams to enable this [32]. Additionally, the organisational literature has identified the importance of teams in the production of knowledge [18,33,34].

Developing structures between and outside health organisations, including the development of research networks seems important for capacity building [12,24,34]. The Department of Health in the UK [14] categorizes this supra-organisational support infrastructure to include centres of academic activity, Research & Development Support Units, and research networks.

As interventions for RCB are targeted at different levels, the framework for measuring its effectiveness mirrors this. However, these levels should not be measured in isolation. One level can have an impact on capacity development at another level, and could potentially have a synergistic or detrimental effect on the other.

### The six principles of research capacity building

Evaluation involves assessing the success of an intervention against a set of indicators or criteria [35,36], which Meyrick and Sinkler [37] suggest should be based on underlying principles in relation to the initiative. For this reason the framework includes six principles of capacity building. The rationale for each principle is given below, along with a description of some suggested criteria for each principle. The criteria presented are not an exhaustive list. As the framework is developed and used in practice, a body of criteria will be developed and built on further.

### Principle 1. Research capacity is built by developing appropriate skills, and confidence, through training and creating opportunities to apply skills

#### Rationale

The need to develop research skills in practitioners is well established [3,4,6], and can be supported through training [14,26], and through mentorship and supervision [15,24,28]. There is some empirical evidence that research skill development increases research activity [23,38], and enhances positive attitudes towards conducting and collaborating in research [39]. Other studies cite lack of training and research skills as a barrier to doing research [30,31]. The need to apply and use research skills in practice is highlighted in order to build confidence [40]and to consolidate learning.

Some needs assessment studies highlight that research skills development should adopt 'outreach' and flexible learning packages and acknowledge the skills, background and epistemologies of the professional groups concerned [7,15,39,41,42]. These include doctors, nurses, a range of allied health professional and social workers. Developing an appropriate mix of professionals to support health services research means that training should be inclusive and appropriate to them, and adopt a range of methodologies and examples to support appropriate learning and experience [15,31,41]. How learning and teaching is undertaken, and the content of support programmes to reflect the backgrounds, tasks and skills of participants should therefore be measured. For example, the type of research methods teaching offered by networks and support units should reflect a range and balance of skills needed for health service research, including both qualitative and quantitative research methods.

Skills development also should be set in the context of career development, and further opportunities to apply skills to practice should be examined. Policy and position statements [14,26] support the concept of career progression or 'careers escalator', which also enables the sustainability of skills. Opportunities to apply research skills through applications for funding is also important [9,10,22,43,44].

At team and network level Fenton et al [34]suggest that capacity can be increased through building intellectual capacity (sharing knowledge), which enhances the ability to do research. Whilst there is no formal measure for this, an audit of the transfer of knowledge would appear to be beneficial. For example teams may share expertise within a project to build skills in novice researchers [45]which can be tracked, and appropriate divisions of workload through reading research literature and sharing this with the rest of the team/network could be noted.

The notion of stepping outside of a safety zone may also suggest increased confidence and ability to do research. This may be illustrated at an individual level by the practitioner-researcher taking on more of a management role, supervising others, or tackling new methodologies/approaches in research, or in working with other groups of health and research professionals on research projects. This approach is supported by the model of RCB suggested by Farmer and Weston [15] which supports progress from participation through to academic leadership.

Some examples of criteria for measuring skills and confidence levels are give in table 1.

**Table 1:** Building skills and confidence

Structural level	Examples of suggested criteria
Individual	• Skills developed (and how) • Evidence of progressive skill development • Evidence of confidence building through sharing new skills with others, applying existing skills in new situations, working with other professional groups in research • Research undertaken
Teams	• Skills developed (and how) • Skill mix of team • Skill/knowledge transfer tracked- (intellectual capital) • Evidence of progressive skill development • Evidence of confidence building through sharing new skills with others, applying existing skills in new situations, working with other professional groups in research • Research undertaken
Organisational	• Evidence of training research needs assessment • Availability and use of training funds • Evidence of outreach work undertaken in organisations • Levels of skills within workforce, and skill mix of the skills across groups • Evidence of matching novice and experienced researchers • Research undertaken, funding approved.
Supra organisational (networks and support units)	• Provision of flexible learning packages • Provision of training shaped around the skills, background and needs of differing professional groups • Examples of knowledge/information transfer (through a variety of mechanisms, including workshops, web-based discussions forums) • Evidence of outreach work, its take up and use • Responses to needs based work • Evidence of secondment opportunities offered and taken up.

### Principle 2. Research capacity building should support research 'close to practice' in order for it to be useful

#### Rationale

The underlying philosophy for developing research capacity in health is that it should generate research that is useful for practice. The North American Primary Care Group [24] defined the 'ultimate goal' of research capacity development as the generation and application of new knowledge to improve the health of individuals and families (p679). There is strong support that 'useful' research is that which is conducted 'close' to practice for two reasons. Firstly by generating research knowledge that is relevant to service user and practice concerns. Many argue that the most relevant and useful research questions are those generated by, or in consultation with, practitioners and services [3,11,24], policy makers [46] and service users [47,48]. The level of 'immediate' usefulness [49] may also mean that messages are more likely to taken up in practice[50]. Empirical evidence suggests that practitioners and policy makers are more likely to engage in research if they see its relevance to their own decision making [31,39,46]. The notion of building research that is 'close to practice' does not necessarily mean that they are small scale, but that the research is highly relevant to practice or policy concerns. A large network of practitioners could facilitate large scale, experimental based projects for example. However, the adoption of certain methodologies is more favoured by practice because of their potential immediate impact on practice [47] and this framework acknowledges such approaches and their relevance. This includes action research projects, and participatory inquiry [31,42]. An example where this more participatory approach has been developed in capacity building is the WeLREN (West London Research Network) cycle [51]. Here research projects are developed in cycles of action, reflection, and dissemination, and use of findings is integral to the process. This network reports high levels of practitioner involvement.

Secondly, building research capacity 'close to practice' is useful because of the skills of critical thinking it engenders which can be applied also to practice decision making [28], and which supports quality improvement approaches in organisations [8]. Practitioners in a local bursary scheme, for example, said they were more able to take an evidence-based approach into their every day practice [9].

Developing a 'research culture' within organisations suggests a closeness to practice that impacts on the ability of teams and individuals to do research. Lester et al [23] touched on measuring this idea through a questionnaire where they explored aspects of a supportive culture within primary care academic departments. This included aspects around exploring opportunities to discuss career progression, supervision, formal appraisal, mentorship, and junior support groups. This may be a fruitful idea to expand further to develop a tool in relation to a health care environment.

Some examples of criteria for measuring the close to practice principle are give in table 2

**Table 2:** Close to practice

Structural level	Examples of suggested criteria
Individuals and teams	• Evidence of clinical expertise and 'hunches' within the research questions and projects • Examples of critical thinking used in practice • Evidence of patient centred outcome measures in projects, and impact of project on patients' quality of life, including social capital and health gain. • Use of methodologies that are action orientated • Use of methodologies that include cost effectiveness approaches • Evidence on level, and nature, of service user involvement
Organisational	• Evidence of informing research questions by gaps in knowledge at an organisational level • Measurements on a culture where research is 'valued, accepted, encouraged and enjoyed'. • Evidence of managerial support/involvement on research projects • Evidence of supporting service user links in research
Supra-organisational (networks and support units)	• Evidence of research questions being developed with practice, needs and priorities • Co-ordination of research programmes between health organisations and university • Development and use of outcomes measures useful for research and practice • Development and use of cost effectiveness methodologies • Action research orientated approaches undertaken • Development of service user panels

### 3. Linkages, partnerships and collaborations enhance research capacity building

#### Rationale

The notion of building partnerships and collaborations is integral to capacity building [19,24]. It is the mechanism by which research skills, and practice knowledge is exchanged, developed and enhanced [12], and research activity conducted to address complex health problems [4]. The linkages between the practice worlds and that of academia may also enhance research use and impact [46].

The linkages that enhance RCB can exist between

• Universities and practice [4,14,43]

• Novice and experienced researchers [22,24,51].

• Different professional groups [2,4,20,34]

• Different health and care provider sectors [4,31,47,52]

• Service users, practitioners and researchers [47,48]

• Researchers and policy makers [46]

• Different countries [28,52]

• Health and industry [53,54]

It is suggested that it is through networking and building partnerships that intellectual capital (knowledge) and social capital (relationships) can be built, which enhances the ability to do research [12,31,34]. In particular, there is the notion that the build up of trust between different groups and individuals can enhance information and knowledge exchange[12]. This may not only have benefits for the development of appropriate research ideas, but may also have benefits for the whole of the research process including the impact of research findings.

The notion of building links with industry is becoming progressively evident within policy in the UK [54] which may impact on economic outcomes to health organisations and the society as a whole[55,56].

Some examples of criteria for measuring linkages and collaborations are given in table 3.

**Table 3:** Linkages, collaborations and partnerships.

Structural level	Examples of suggested criteria
Individual	• Who they have worked with: to gain knowledge and to share knowledge • Evidence of increased number of research partnerships • Evidence of inter-professional working
Teams	• Who the team has worked with: academic and practice • Network development (work with other teams) • Evidence of inter-professional and other links
Organisational	• Links with universities/RDSUs • Evidence of joint posts with university • Evidence of working with other service organisations on research • Evidence of contribution/memberships to Networks • Work with funding bodies
Supra-organisational (networks and support units)	• Joint posts hosted • Evidence of research collaboration with practitioners, teams, networks and organisations in health care practice • Development of links across networks • International links

### 4. Research capacity building should ensure appropriate dissemination to maximize impact

#### Rationale

A widely accepted measure to illustrate the impact of RCB is the dissemination of research in peer reviewed publications, and through conference presentations to academic and practice communities [5,12,26,57]. However this principle extends beyond this more traditional method of dissemination. The litmus test that ultimately determines the success of capacity building is that it should impact on practice, and on the health of patients and comminutes[24] that is; the social impact of research [25]. Smith [25]argues that the strategies of dissemination should include a range of methods that are 'fit for purpose'. This includes traditional dissemination, but also includes other methods, for example, instruments and programmes of care implementation, protocols, lay publications, and publicity through factsheets, the media and the Internet.

Dissemination and tracking use of products and technologies arising from RCB should also be considered, which relate to economic outcomes of capacity building [55]. In the UK, the notion of building health trusts as innovative organisations which can benefit economically through building intellectual property highlights this as an area for potential measurement [56].

Some examples of criteria for measuring appropriate dissemination are given in table 4

**Table 4:** Appropriate dissemination and impact

Structural level	Examples of suggested criteria
Individuals and Teams	• Papers in research and practice journals • Conference presentations • Applied dissemination of findings • Evidence of influence on local strategy and planning
Organisational	• Ease of access to research undertaken locally • Seminar programmes relating to research undertaken • Examples of evidence based practice and applying locally developed knowledge in strategy policy and practice • Funding to support practitioners and teams to disseminate findings • Successful applications for intellectual property submitted based on R&D developed in organisation
Supra-organisational (networks and support units)	• Papers focussing on health services research, written with practitioners • Conference presentations at practice- focussed conferences • Applied dissemination • Innovative dissemination • Successful applications for intellectual property submitted based on R&D developed in partnership with health organisations

### 5. Research capacity building should include elements of continuity and sustainability

#### Rationale

Definitions of capacity building suggest that it should contain elements of sustainability which alludes to the maintenance and continuity of newly acquired skills and structures to undertake research [18,19]. However the literature does not explore this concept well [19]. This in itself may be partly due problems around measuring capacity building. It is difficult to know how well an initiative is progressing, and how well progress is consolidated, if there are no benchmarks or outcomes against which to demonstrate this.

Crisp et al [19] suggests that capacity can be sustained by applying skills to practice. This gives us some insight about where we might look for measures of sustainability. It could include enabling opportunities to extend skills and experience, and may link into the concept of a career escalator. It also involves utilizing the capacity that has been already built. For example engaging with those who have gained skills in earlier RCB initiatives to help more novice researchers, once they have become 'experts', and in finding an appropriate place to position the person with expertise with the organisation. It could also be measured by the number of opportunities for funding for continued application of skills to research practice.

Some examples of criteria for measuring sustainability and continuity are gibe in table 5

**Table 5:** Continuity and sustainability

Structural level	Examples of suggested criteria
Individual	• Successful access to funding for continued application of skills (grants and fellowships) • Continued contacts with collaborators/linkages • Examples of continued support and supervision arrangements
Teams	• Recognition and matching of skills • Successful access to funding for continued application of skills
Organisational	• Secondment opportunities, available and used • Local responsive funding access and use • Recognition and matching of skills • Examples of continued collaboration
Supra-organisational (networks and support units)	• Examples of continued collaboration • Linked support within career pathways • Fellowships supported

### 6. Appropriate infrastructures enhance research capacity building

#### Rationale

Infrastructure includes structures and processes that are set up to enable the smooth and effective running of research projects. For example, project management skills are essential to enable projects to move forward, and as such should be measured in relation to capacity building. Similarly, projects should be suitably supervised with academic and management support. To make research work 'legitimate' it may be beneficial to make research a part of some job descriptions for certain positions, not only to reinforce research as a core skill and activity, but also to review in annual appraisals, which can be a tool for research capacity evaluation. Information flow about calls for funding and fellowships and conferences is also important. Hurst [42] found that information flow varied between trusts, and managers were more aware of research information than practitioners.

The importance of protected time and backfill arrangements as well as funding to support this, is an important principle to enable capacity building [9, 15, 24, 58]. Such arrangements may reduce barriers to participation and enable skills and enthusiasm to be developed[15]. Infrastructure to help direct new practitioners to research support has also been highlighted[14]. This is particularly true in the light of the new research governance and research ethics framework in the UK [59]. The reality of implementing systems to deal with the complexities of the research governance regulations has proved problematic, particularly in primary care, where the relative lack of research management expertise and infrastructure has resulted in what are perceived as disproportionately bureaucratic systems. Recent discussion in the literature has focused on the detrimental impact of both ethical review, and NHS approval systems, and there is evidence of serious delays in getting research projects started [60]. Administrative and support staff to help researchers through this process is important to enable research to take place [61].

Some examples of criteria for measuring are given in table 6.

**Table 6:** Infrastructure

Structural level	Examples of suggested criteria
Individual	• Evidence of project management in projects (objective setting with time scales) • A description of mentorship and supervision structures • Research is part of job description and reviewed in annual appraisal
Teams	• Evidence of project management in projects • A description of mentorship and supervision • Protected time taken
Organisational	• Evidence of R&D information dissemination strategies • Use and availability of protected time • Evidence of back fill availability and use • Research is part of annual appraisal for some jobs • Evidence of help with governance and ethics
Supra-organisational (networks and support units)	• The nature of collaborations (co-authorship, order of authorship) • Organize information exchange events. Description of attendance

## Conclusion

This paper suggests a framework which sets out a tentative structure by which to start measuring the impact of capacity building interventions, and invites debate around the application of this framework to plan and measure progress. It highlights that interventions can focus on individuals, teams, organisations, and through support infrastructures like RDSUs and research networks. However, capacity building may only take place once change has occurred at more than one level: for example, the culture of an organisation in which teams and individuals work may have an influence of their abilities and opportunities to do research work. It is also possible that the interplay between different levels may have an effect on the outcomes at other levels. In measuring progress, it should be possible to determine a greater understanding of the relationship between different levels. The framework proposed in this paper may be the first step to doing this.

The notion of building capacity at any structural level is dependent on funding and support opportunities, which are influenced by policy and funding bodies. The ability to build capacity across the principles developed in the framework will also be dependent of R&D strategy and policy decisions. For example, if policy fluctuates in its emphasis on building capacity 'by', 'for' or 'with' practice, the ability to build capacity close to practice will be affected.

In terms of developing a science of RCB, there is a need to capture further information on issues of measuring process and outcome data to understand what helps develop 'useful' and 'useable' research. The paper suggests principles whereby a number of indicators could be developed. The list is not exhaustive, and it is hoped that through debate and application of the framework further indicators will be developed.

An important first step to building the science of RCB should be debate about identifying appropriate outcomes. This paper supports the use of traditional outcomes of measurement, including publications in peer reviewed journals and conference presentations. This assures quality, and engages critical review and debate. However, the paper also suggests that we might move on from these outcomes in order to capture the social impact of research, and supports the notion of developing outcomes which measure how research has had an impact on the quality of services, and on the lives of patients and communities. This includes adopting and shaping the type of methodologies that capacity building interventions support, which includes incorporating patient centred outcomes in research designs, highlighting issues such as cost effectiveness of interventions, exploring economic impact of research both in terms of product outputs and health gain, and in developing action oriented, and user involvement methodologies that describe and demonstrate impact. It also may mean that we have to track the types of linkages and collaborations that are built through RCB, as linkages that are close to practice, including those with policy makers and practitioners, may enhance research use and therefore 'usefulness'. If we are to measure progress through impact and change in practice, an appropriate time frame would have to be established alongside these measures.

This paper argues that 'professional outcomes' should also be measured, to recognize how critical thinking developed during research impacts on clinical practice more generally.

Finally, the proposed framework provides the basis by which we can build a body of evidence to link process to the outcomes of capacity building. By gathering process data and linking it to appropriate outcomes, we can more clearly unpack the 'black box' of process, and investigate which processes link to desired outcomes. It is through adopting such a framework, and testing out these measurements, that we can systematically build a body of knowledge that will inform the science and the art of capacity building in health care.

## Summary

• There is currently little evidence on how to plan and measure progress in research capacity building (RCB), or agreement to determining its ultimate outcomes.

• Traditional outcomes of publications in peer reviewed journals, and successful grant applications may be the easy and important outcomes to measure, but do not necessarily address issues to do with the usefulness of research, professional outcomes, the impact of research activity on practice, or on measuring health gain.

• The paper suggests a framework which provides a tentative structure by which measuring the impact of RCB could be achieved, shaped around six principles of research capacity building, and includes four structural levels on which each principle can be applied.

• The framework could be the basis by which RCB interventions could be planned, and progress measured. It could act as a basis of comparison across interventions, and could contribute to establishing a knowledge base on what is effective in RCB in healthcare

## Competing interests

The author(s) declare that they have no competing interests.

## Pre-publication history

The pre-publication history for this paper can be accessed here:


